# Peptides Derived from HIV-1 Integrase that Bind Rev Stimulate Viral Genome Integration

**DOI:** 10.1371/journal.pone.0004155

**Published:** 2009-01-07

**Authors:** Aviad Levin, Zvi Hayouka, Markus Helfer, Ruth Brack-Werner, Assaf Friedler, Abraham Loyter

**Affiliations:** 1 Department of Biological Chemistry, The Alexander Silberman Institute of Life Sciences, Hebrew University of Jerusalem, Jerusalem, Israel; 2 Institute of Chemistry, The Hebrew University of Jerusalem, Jerusalem, Israel; 3 Institute of Virology, Helmholtz Center Munich - German Research Center for Environmental Health, Ingolstaedter Landstr, Neuherberg, Germany; 4 Clinical Cooperation Group ‘Immune-Monitoring’, Helmholtz Center Munich - German Research Center for Environmental Health, Ingolstaedter Landstr, Neuherberg, Germany; AIDS Research Center, Chinese Academy of Medical Sciences and Peking Union Medical College, China

## Abstract

**Background:**

The human immunodeficiency virus type 1 (HIV-1) integrase protein (IN), catalyzes the integration of viral DNA into the host cell genome. IN catalyzes the first step of the integration process, namely the 3′-end processing in which IN removes a pGT dinucleotide from the 3′ end of each viral long terminal repeat (LTR). Following nuclear import of the viral preintegration complex, the host chromosomal DNA becomes accessible to the viral cDNA and the second step of the integration process, namely the strand-transfer step takes place. This ordered sequence of events, centered on integration, is mandatory for HIV replication.

**Methodology/Principal Findings:**

Using an integrase peptide library, we selected two peptides, designated INr-1 and INr-2, which interact with the Rev protein and probably mediate the Rev-integrase interaction. Using an *in-vitro* assay system, we show that INr-1 and INr-2 are able to abrogate the inhibitory effects exerted by Rev and Rev-derived peptides on integrase activity. Both INr-1 and INr-2 were found to be cell-permeable and nontoxic, allowing a study of their effect in HIV-1-infected cultured cells. Interestingly, both INr peptides stimulated virus infectivity as estimated by production of the viral P24 protein, as well as by determination of the appearance of newly formed virus particles. Furthermore, kinetics studies revealed that the cell-permeable INr peptides enhance the integration process, as was indeed confirmed by direct determination of viral DNA integration by real-time PCR.

**Conclusions/Significance:**

The results of the present study raise the possibility that in HIV-infected cells, the Rev protein may be involved in the integration of proviral DNA by controlling/regulating the activity of the integrase. Release from such inhibition leads to stimulation of IN activity and multiple viral DNA integration events.

## Introduction

The human immunodeficiency virus type 1 (HIV-1) integrase protein (IN), which is part of the Gag-Pol precursor, catalyzes the integration of viral DNA into the host cell genome. It is contained in the virion and following infection, it is released into the cytoplasm of infected cells [1]. After a reverse-transcription step [2], IN becomes part of the preintegration complex (PIC) which also includes the newly obtained viral cDNA, as well as the viral matrix, Vpr, and nucleocapsid proteins [1]–[8]. Within the cytoplasm, IN catalyzes the first step of the integration process, namely the 3′-end processing in which an IN dimer removes a pGT dinucleotide from the 3′ end of each viral long terminal repeat (LTR) [9], [10]. Following nuclear import of the PIC, the host chromosomal DNA becomes accessible to the viral cDNA and the second step of the integration process, namely the strand-transfer step that is catalyzed by an IN tetramer, takes place [9], [11]–[13]. This ordered sequence of events, centered on integration, is mandatory for HIV replication. Within the nuclei of the infected cells, the cellular protein, lens epithelium-derived growth factor (LEDGF)/p75, as well as some other cellular cofactors assist the integration process by tethering the IN to the host chromosomal DNA [14], [15].

Although each HIV-1 infected cell contains several copies of the viral genome, only a limited number of integration events per cell, mostly one or two, have been observed [16], [17]. On the other hand, in cells infected by other retroviruses such as murine leukemia virus (MuLV) or Rous sarcoma virus (RSV), numerous integration events per cell have been detected [18]–[21]. This suggests that control of integration is different for HIV-1 thanfor other retroviruses.

Recently, using bimolecular fluorescence complementation (BiFC) and coimmunoprecipitation assay systems, we have shown that the HIV-1 IN and Rev proteins can interact with each other intracellularly [22].

Based on these results [22], we speculate that the limited number of integration events observed in HIV-infected cells may result from regulated inhibition of IN enzymatic activity by the viral Rev protein.

The karyophilic Rev protein, whose expression is promoted by viral DNA, is required at the late phase of the viral replication cycle for promoting nuclear export of partially spliced or un-spliced viral RNA [23], [24]. Rev nuclear export is crucial for Rev-dependent activation of HIV gene expression [25]. In addition, Rev selectively activates the production of structural HIV components, including the HIV genome itself [24], [26], [27]. Interestingly, a few studies have clearly indicated that several viral proteins—among them the Rev protein—can be transcribed from nonintegrated viral DNA, the amount of which can reach up to 99% of the total viral DNA present in infected cells [28], [29]. The Rev protein is thus also expressed at early stages of virus infection, probably before the integration step occurs [17], [30]–[32]. The possibility that the Rev protein observed before the integration step may arrive with the virus particles cannot be eliminated. However the Rev protein was not, so far, reported to be present in virus particles or in the PIC [1], [3]–[7].

Following our previously described Rev-IN interaction [22], we identified two short Rev-derived peptides' derived from residues 13–23 and 53–67 of Rev, that inhibit IN enzymatic activities *in vitro* and HIV-1 replication in cultured cells [22]. Similar to the Rev-derived peptides also the Rev protein—as demonstrated in the present work—is able to inhibit the enzymatic activity of IN.

In the present work we demonstrate that the Rev-mediated inhibitory effect, as well as that exerted by the Rev 13–23 and Rev 53–67 peptides, can be abrogated by two selected IN-derived peptides, designated INr-1 and INr-2, due to displacement of the inhibitory molecules. Furthermore INr-1 and INr-2, which have been found to be cell-permeable, stimulated integration of the HIV-1 cDNA into host chromosomal DNA. Based on the results obtained in the *in-vitro* assay systems, it is conceivable that the effects seen in cells also resulted from dissociation of a putative IN-Rev complex by these peptides. Thus, our present results further support the view that intracellularly, the viral Rev protein may inhibit/regulate IN activity.

## Results

### A) Selection of IN-derived peptides that interact with the Rev protein and Rev-derived peptides

**Figure 1:**
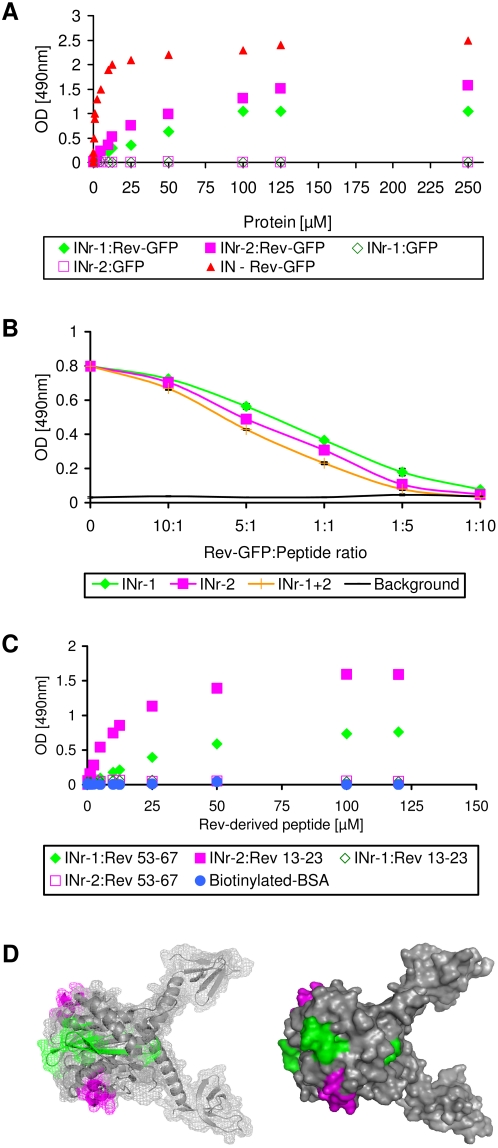
INrs interact with Rev-GFP and Rev-derived peptides in vitro and promote dissociation of Rev-IN complexes. (A) Rev-GFP or GFP alone were incubated in ELISA plates coated with either INr-1 (Green full and empty diamond respectively) or INr-2 (Magenta full and empty square respectively) and binding was determined as described in Materials and Methods. IN-Rev-GFP interaction was used as positive control (Red triangle) (B) The IN protein was first bound to the ELISA plate and then incubated with Rev-GFP to obtain a Rev-IN complex. The complex was then incubated with either one or both of the INr peptides at the designated Rev-GFP∶peptide ratio. Wells were then washed and the amount of bound Rev-GFP was determined by mouse anti-GFP antibody and secondary rabbit anti-mouse HRP-conjugated antibody. (C) Rev 13–23 and Rev 53–67 (conjugated to biotinylated-BSA) were incubated in ELISA plates coated with either INr-1 (Green empty and full diamond respectively) or INr-2 (Magenta full and empty square respectively) and binding was estimated as described in Materials and Methods. All other experimental conditions were as described previously [37], [55] and in Materials and Methods. (D) Localization of INr-1 (green) and INr-2 (magenta) peptides within the IN protein was determined using a protein model (PDB: 1EX4 [35]). A clear model is shown on the left to illustrate the exact domains of INr-1 and INr-2 within the IN protein. Both models, left and right, emphasize exposure of the INr sequences on the protein surface. The models presented in this figure were generated using PyMOL v0.99 software (DeLano Scientific LLC).

In order to further characterize the previously described Rev-IN interaction [22], attempts were made, in the present work, to identify the IN domains that mediate this interaction. Screening of an IN peptide library (NIH AIDS Research and Reference Reagent Program, Division of AIDS, NIAID, NIH: HIV-1 Consensus B Pol (15-mer) peptides – Complete Set from DAIDS, NIAID) by an ELISA-based assay system (not shown) revealed that two IN-derived peptides specifically interact with Rev-GFP conjugates. These two peptides were designated as INr-1 and INr-2 and their binding pattern and amino acids sequences are shown Fig 1A and Table 1 respectively. The Rev protein itself was highly insoluble under our experimental conditions [33] and therefore could not be used: instead, we used a Rev-GFP conjugate that is soluble and functional [34]. Specific interaction with the Rev protein was inferred from the results showing that there is no such observable interaction with GFP alone. The results in Fig 1B show that addition of INr-1 and INr-2 peptides to the complex formed between IN and Rev-GFP induces the release of the bound Rev-GFP. This observation further confirmed that the sequences of the INr peptides within the IN protein mediate the interaction with the Rev protein. INr-1 and INr-2 also interacted with the Rev 53–67 and Rev 13–23 peptides, respectively (Fig 1C and Table 1 and 2). This was inferred from experiments showing that biotinylated-BSA-Rev 13–23 or Rev 53–67 conjugates were able to interact with INr peptides-coated plates (Fig 1C). No binding was observed with biotinylated-BSA alone (Fig 1C), indicating specific binding to the INr peptides. The same specific interaction was observed when the order of addition was reversed, namely, when soluble biotinylated-BSA-INr conjugates were incubated with plates coated with Rev 13–23 and Rev 53–67 (not shown). As is evident from the results in Fig 1C, INr-1 interacted with Rev 53–67 peptide but not with the Rev 13–23 peptide, while INr-2 interacted with Rev 13–23 but not with Rev 53–67, indicating specificity of interaction. Binding of the INrs to the Rev-derived peptides showed somewhat higher affinity than to the Rev protein itself, as indicated by their apparent *K_d_*s (Table 2).

**Table 1:** Interaction of IN peptides derived from an IN peptide library, with Rev-GFP or Rev-derived peptides.

NIH Cat #	Amino acid residues within the IN	Sequence*	Peptide designated name	Interacting with
5656	66–80	WTHLEGKIILVAVHVA	INr-1	Rev 53–67, Rev-GFP
5669	118–128	WGSNFTSTTVKA	INr-2	Rev 13–23, Rev-GFP

The ELISA binding system was used to screen for interactions between the IN peptide library and Rev-GFP protein as well as between the library and the two (inhibitory) Rev-derived peptides (Fig 1).

* A tryptophan was added at the N' terminus of the peptides in order to determine their concentrations.

For experimental details see Materials and Methods and Fig 1.

**Table 2:** Quantitative characterization of the interaction between the INrs and Rev-GFP or Rev-derived peptides.

Interaction between	Apparent *K_d_* [µM]
INr-1 : Rev-GFP	41±6
INr-1 : Rev 53–67 - Bb	23±1
INr-2 : Rev-GFP	27±1
INr-2 : Rev 13–23 - Bb	11±1

Apparent *K_d_* of the interaction was obtained by the ELISA-based system and their values were calculated as described in [64].

For experimental details see Materials and Methods and Fig 1.

Examination of the 3D structure of a truncated IN (aa52-288, [35], PDB: 1EX4) revealed that the INr peptides are derived from the surface-exposed region of the IN (Fig 1D).

### B) Inhibition of the IN enzymatic activity by the Rev protein and Rev-derived peptides: specific abrogation of inhibition by the INr peptides

**Figure 2:**
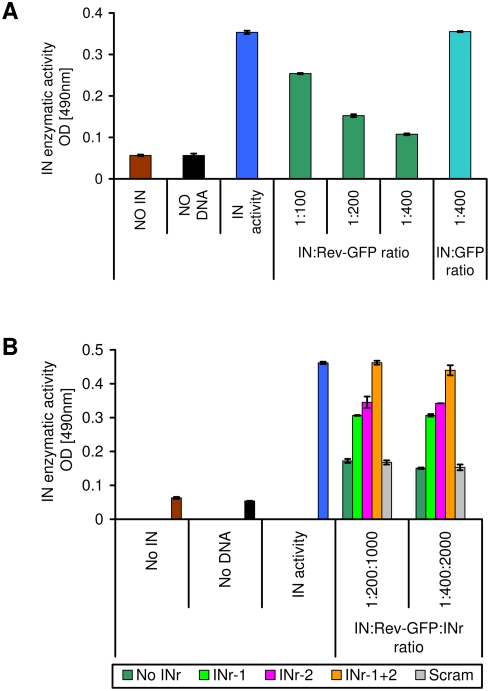
Inhibition of IN enzymatic activity by Rev and its abrogation by the INr peptides. (A) Rev-GFP (dark green) or GFP (turquoise) were incubated at different molar ratios with IN (390 nM) and the IN enzymatic activity was determined as described previously [22] and in Materials and Methods. (B) Rev-GFP was preincubated with the indicated INr peptides at a molar ratio of 5∶1 (peptide∶Rev-GFP) and the resultant mixture was added to a solution containing IN to give a molar ratio of 1∶200 and 1∶400 (IN∶Rev-GFP). Following an incubation period, IN activity was estimated as described previously [22]. All other experimental conditions are described in Materials and Methods.

**Figure 3:**
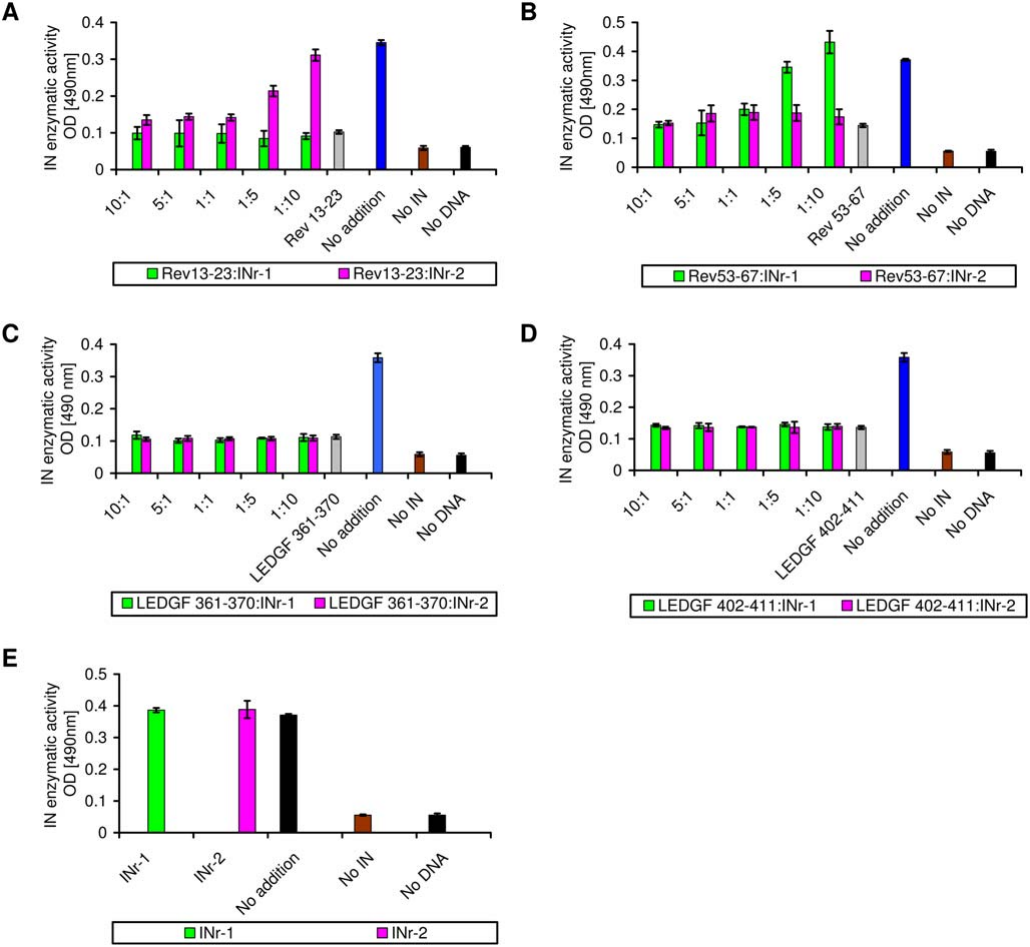
Inhibition of IN activity by Rev- and LEDGF-derived peptides: specific abrogation by the INr peptides. IN (390 nM) was incubated with 50 µM of the Rev-derived peptides (1∶300 IN∶Rev-derived peptide) [22] (Rev13–23 (A) and Rev 53–67 (B)) or LEDGF-derived peptides [36] (LEDGF 361–370 (C)) and LEDGF 402–411 (D)) and then the INr peptides were added at different molar ratios ranging from 10∶1 to 1∶10 (Rev or LEDGF peptide∶INr peptide). (E) IN (390 nM) was incubated with the INr peptides (50 µM).

The results depicted in Fig 2A clearly show that the enzymatic activity of IN is inhibited by Rev-GFP due to specific interaction with the Rev protein itself and not with the GFP (Fig 2A). At a Rev-GFP∶IN (mole/mole) ratio of about 100, approx. 30% inhibition was already observed, reaching up to 70% inhibition at a ratio of 400 (Fig 2A). Interestingly, the INr peptides abrogated the inhibitory activities of Rev-GFP (Fig 2B). The results in Fig 3A confirm previously described results [22] showing that the Rev-derived peptides (Rev 53–67 and Rev 13–23 blocked IN enzymatic activity, reaching 60 to 70% inhibition at a peptide∶IN (mol/mol) ratio of about 150 (Fig 3A and B). It is also evident that the INr peptides were able to abrogate the inhibitory effect of the Rev derived peptides on integrase activity. Integrase activity was fully restored in the presence of both INr peptides at a molar ratio of Rev∶INr peptides of 1∶5–1∶10 (Fig 3A and B)). The fact that no abrogation was observed when the Rev∶INr peptides ratios was 1∶1 can be explained by the affinity differences towards their binding partners ([22] and table 2). This appeared to be due to the ability of the INrs to specifically interact with Rev-GFP and the Rev-derived peptides, since incubation with a scrambled peptide did not restore the integrase activity (Fig. 3B).

As expected from their interaction specificity (see Table 1) INr-1 abrogated the inhibitory effect of the Rev 53–67 peptide while INr-2 that exerted by the Rev 13–23 peptide (fig 3A and B)

The INrs did not, by themselves, have any effect on IN enzymatic activity (Fig 3E) or on Rev protein activity which was estimated by a previously described method [25] (not shown). Further evidence for the specific effect of the INrs in releasing the observed inhibition of IN enzymatic activity can be inferred from the lack of effect of a scrambled peptide (Fig 2B). As mentioned above and described before in addition to the inhibitory Rev derived peptides we also have identified two LEDGF peptides which blocked the IN enzymatic activity [36]. However the INrs failed to abrogate the inhibitory effect exerted on IN by LEDGF-derived inhibitory peptides and Fig 3C and D), again emphasizing the specific interaction between the INrs and the Rev protein and peptides.

### C) Inhibition of HIV-1 infection by Rev 53–67 and Rev 13–23 peptides in MAGI cultured cells: specific abrogation of inhibition by INr peptides

**Figure 4:**
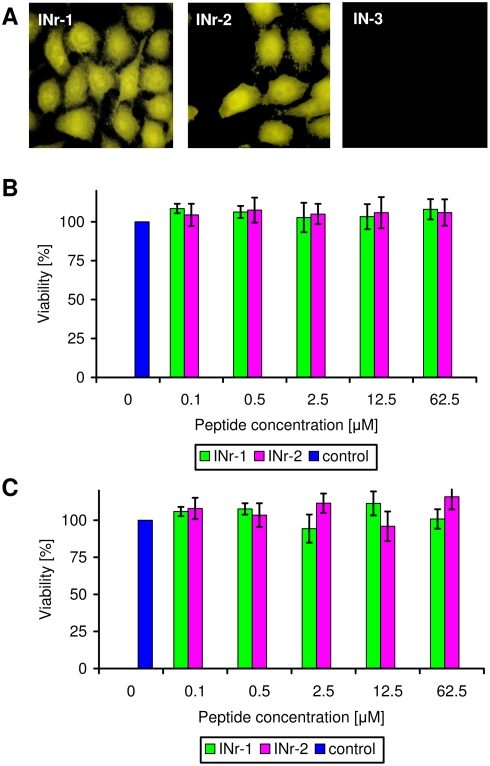
Cell penetration and toxicity of INr peptides. (A) Fluorescein-labeled INr-1, INr-2, and IN-3 (a known nonpermeable peptide [37]) each at 10 µM, were incubated for 2 h at 37°C with HeLa cells. The cells were then washed three times with PBS and visualized by confocal microscopy. (B) Cell toxicity was determined by MTT assay in HeLa TZM-bl cells. (C) Same as (B) but in H9 lymphocytes. Experimental conditions are described in Materials and Methods.

It is evident from the results shown in Fig 4A that both INr-1 and INr-2 are cell-permeable peptides. Our results (Fig 4A) showing that another peptide of about the same size, designated IN-3 (see [37]), failed to penetrate the cultured HeLa cells clearly indicate the integrity of the cells' plasma membranes as well as their viability (see also Fig 4B and C). No toxic effect was observed when both peptides were incubated, up to about 62 µM, with either HeLa TZM-bl cells (Fig 4B) or H9-T-cell lymphocytes (Fig 4C). These results allowed us to study their effect on HIV-1 infection in cultured cells.

**Figure 5:**
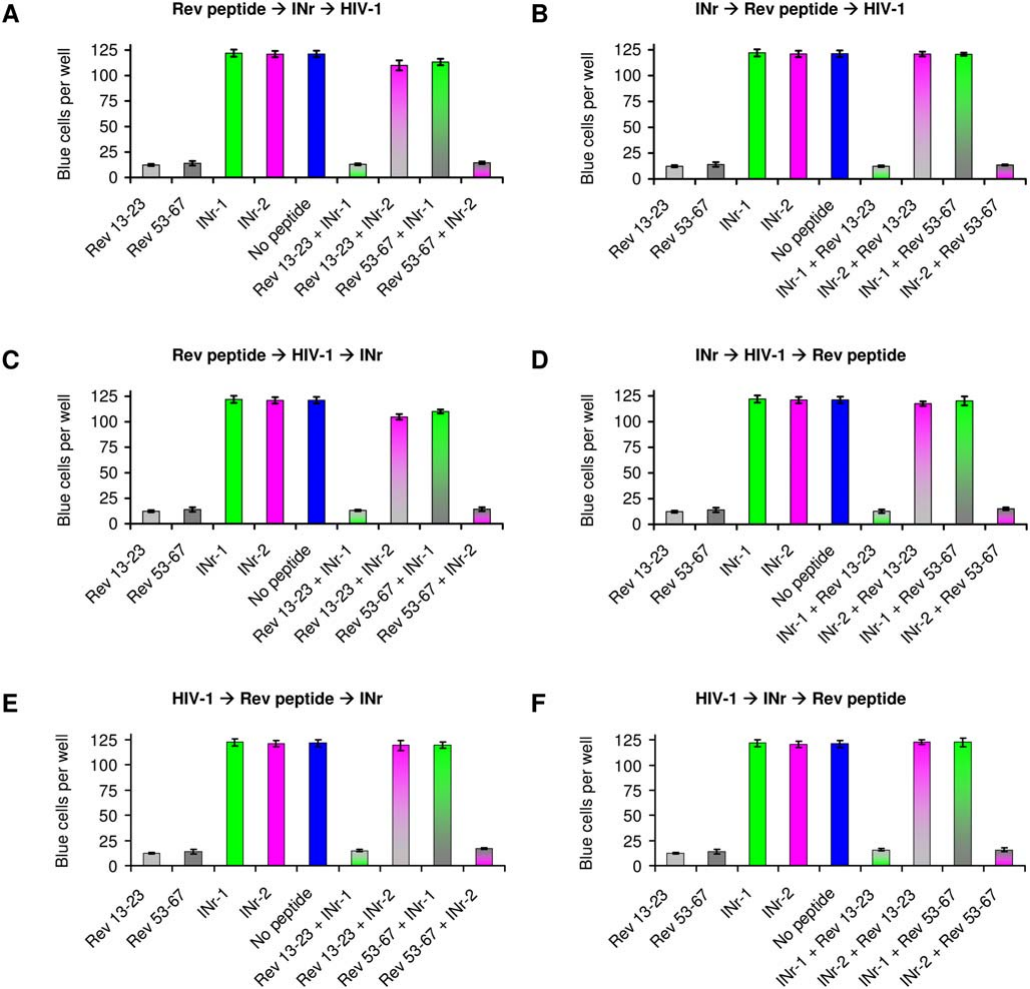
Inhibition of HIV-1 infection by the Rev-derived peptides and abrogation of inhibition by INrs. HeLa MAGI TZM-bl cells were incubated with Rev-derived (12.5 µM of Rev 13–23 or Rev 53–67) and INr (62.5 µM of INr-1 or INr-2) peptides and infected with HIV-1 Delta-env/VSV-G. at different orders of addition as indicated by arrows, A) Rev peptide>INr>HIV-1; (B) INr>Rev peptide>HIV-1; (C) Rev peptide>HIV-1>INr; (D) INr>HIV-1>Rev peptide; (E) HIV-1>Rev peptide>INr; (F) HIV-1>INr>Rev peptide. Control cells were incubated with none or only one of the indicated peptides. The length of the incubation period with each of the above-described components (HIV-1, Rev and INr peptides) was 2 h at 37°C and at the end of the total 6-h incubation, the infected cells were left for a further 48-h incubation. At the end of the incubation period, the number of blue cells per well was estimated as described in Materials and Methods. (A summary of the results is presented in Table 3).

The results in Fig 5A-F and Table 3 show that the Rev-derived peptides 13–23 and 53–67 were able to block HIV-1 infection in cultured cells (see also ref [22]). This is in addition to their ability to inhibit HIV-1 IN activity *in vitro* (see Fig 3). It was, therefore, of interest to determine whether the cell-permeable nontoxic INr peptides, similar to their effect *in vitro*, would be able to abrogate the inhibitory effect of the Rev-derived peptides in cells.

**Table 3:** Inhibition of HIV-1 infection by the Rev-derived peptides and its abrogation by the INr peptides are not affected by order of addition: a summary of the results described in Figure 5.

Peptides	Rev peptide	INr	Rev peptide	INr	Infection	Infection
	↓	↓	↓	↓	↓	↓
	INr	Rev peptide	Infection	Infection	Rev peptide	INr
	↓	↓	↓	↓	↓	↓
	Infection	Infection	INr	Rev peptide	INr	Rev peptide
Rev 13–23+INr-1	13±1	12±1	13±1	13±2	15±1	16±1
Rev 13–23+INr-2	110±5	121±2	105±3	118±2	119±5	123±2
Rev 53–67+INr-1	113±3	121±1	110±2	120±4	119±3	123±4
Rev 53–67+INr-2	15±1	14±1	14±2	15±1	17±1	16±2
Rev 13–23	12±1	12±1	12±1	12±1	12±1	12±1
Rev 53–67	14±2	14±2	14±2	14±2	14±2	14±2
INr-1	122±3	122±3	122±3	122±3	122±3	122±3
INr-2	121±3	121±3	121±3	121±3	121±3	121±3
No peptide	121±3	121±3	121±3	121±3	121±3	121±3

The numbers presented are blue Magi TZM-bl cells/well.

As can be seen (Fig 5), the INrs were able to completely abrogate the ability of the Rev peptides to inhibit HIV infection, regardless of their order of addition. The same abrogation was obtained when the INrs were added either before or after infection with HIV-1 or incubation with the Rev peptides (for details see legend to Fig 5). The results showing that incubation with the Rev peptides blocked HIV-1 infection by more than 85% (Fig 5) strongly indicate the formation of an intracellular nonactive IN-Rev peptide complex. This inhibition was completely reversed by the addition of INr peptides (INr∶Rev molar ratio of 5∶1) regardless of the order of addition (Fig 5). The most interesting observation was that abrogation could be obtained even when the INr peptides were added as the last component, namely, after 4 h of incubation with HIV-1 and 2 h incubation with the Rev peptides (Fig 5E). The same results were obtained following 4 h incubation with the Rev peptides and 2 h with HIV-1 (Fig 5C). In both cases, abrogation can be explained by INr-induced dissociation of the nonactive complex formed between the Rev peptides and the IN protein. As expected (see also Fig 3), INr-1 blocked the inhibitory effect of Rev 53–67 but not of Rev 13–23, while INr-2 had the same effect on Rev 13–23 but not on Rev 53–67, again indicating specificity of interaction (Fig 5). Needless to say, that the INr peptides did not have any effect by themselves on HIV infection in MAGI HeLa cells (Fig 5). The results depicted in Fig 5 are summarized in Table 3.

### D) INr peptides stimulate virus production and integration of the HIV-1 genome in cultured cells

**Figure 6:**
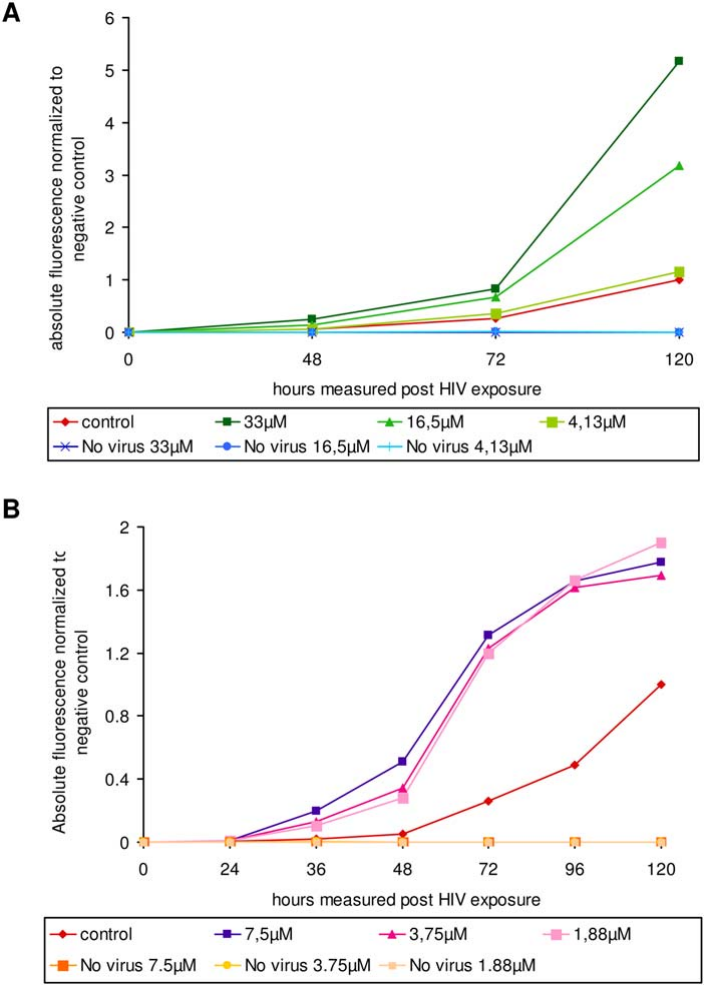
INr peptides stimulate HIV-infection of LC5-RIC cultures. LC5-RIC reporter cells contain an integrated reporter gene for HIV-dependent production of a red fluorescent protein. LC5-RIC cultures were incubated with HIV-1 IIIB and peptide INr-1 (A) or INr-2 (B) at the indicated concentrations and the fluorescent intensities of the cultures measured after the indicated time periods. The graph shows the overall fluorescent intensities measured in peptide-containing cultures relative to HIV-exposed cultures incubated without peptide (solvent only) for the same time period.

**Figure 7:**
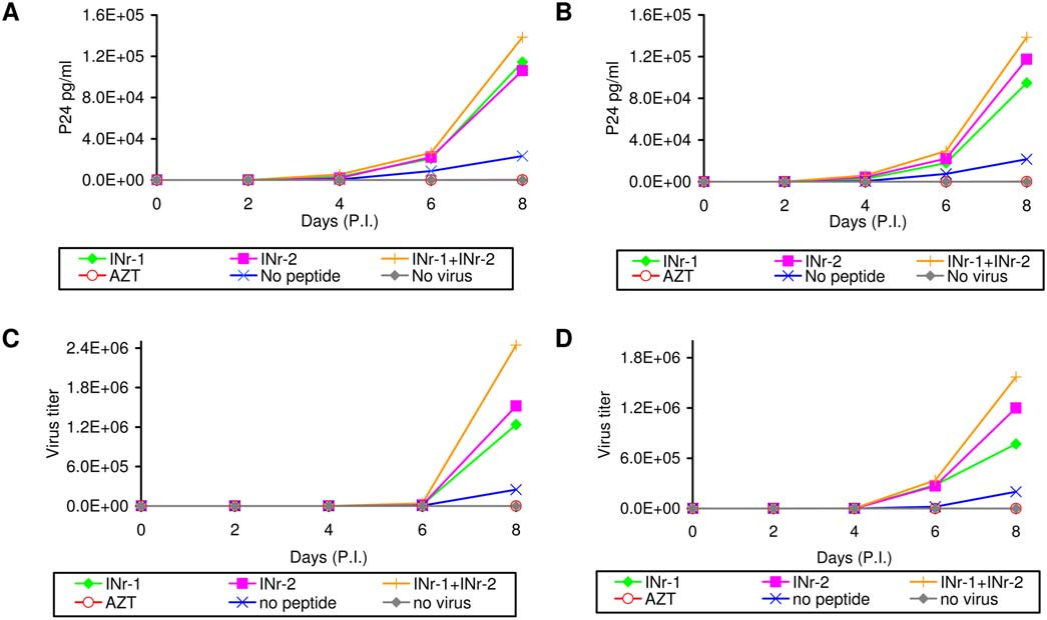
Stimulation of HIV-1 replication by the INrs. The INrs (12.5 µM) were incubated with H9 cells (A and C) and Sup-T1 lymphocyte cells (B and D) which were then infected with HIV-1 (MOI 0.01) as described in Materials and Methods. The amount of viral P24 (A and B) and virus titer (C and D) were determined every 2 days by ELISA and MAGI assay [49], respectively. All other experimental conditions are described in Materials and Methods. The concentration of AZT used was 2 µM.

The results in Fig 6 show that preincubation of the INr peptides with LC5-RIC cells prior to the addition of HIV-1 resulted in significant stimulation of the infection efficiency, as estimated by the expression of a fluorescence reporter gene [25], [38], [39]. Under the experimental conditions used about seven fold stimulation has been obtained with 33 µM of INr-1 following 120 hrs of infection (Fig 6A). About the same stimulation of infection was observed by 7.5 µM of INr-2 but at 72–96 hrs post infection (Fig 6B). The difference in the period required for obtaining maximal stimulation between INr-1 and 2 may be due to a difference in the stability and the activities (see Figs 2) of these two peptides. The decrease in the stimulation degree observed at longer times with INr-2 probably results from an increase in cell death and syncytia formation induced by the combination of the peptide and the virus (not shown). Stimulation of infection by the INr peptides was also observed when it was assayed by the appearance of the viral p24 antigen in H9 and Sup-T1 cultured cells (Fig 7A and B). The same stimulation was also observed when the efficiency of infection was determined by the appearance of infectious viral particles (Fig 7C and D). Stimulation of the infection was slightly enhanced when a mixture of INr-1 and INr-2 was incubated with the cells (Fig 7).

**Figure 8:**
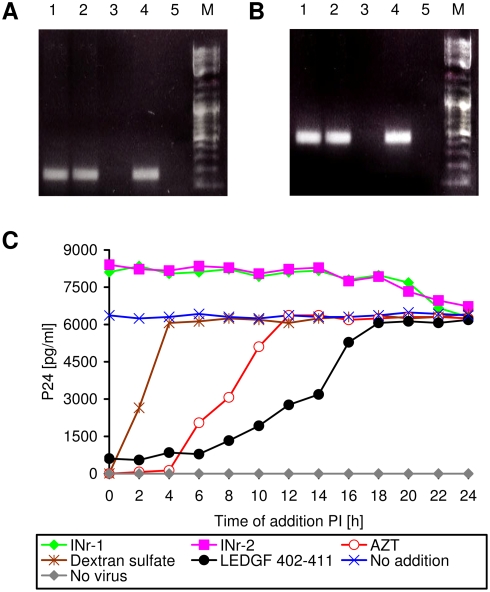
Virus-cell adsorption and viral reverse-transcriptase activity are not affected by the INr peptides. (A and B) Estimation of viral DNA in virus-infected cells: Sup-T1 cells were incubated with the indicated peptides or with AZT for 2 h and then were infected with HIV. Following another 6 h incubation, the viral Gag (A) or Nef (B) DNA sequences were amplified using specific primers. The reaction was terminated after 30 cycles. Lanes: 1) INr-1 (12.5 µM); 2) INr-2 (12.5 µM); 3) AZT (2 µM); 4) untreated cells; 5) uninfected cells. Note that in the AZT-treated cells, reverse-transcribed viral DNA is absent. (C) Influence of the time of addition on P24 production [59]: Sup-T1 cells were infected with HIV-1 at a MOI of 2, and the indicated constituents were added at different time points PI (0, 2, 4,…, 24 h). Viral p24 was determined at 48 h PI. No addition (Blue X); no virus (Gray diamond); dextran sulfate 20 µM (Brown asterisk); AZT 2 µM (Red empty circle); INr-1 12.5 µM (Green diamond); INr-2 12.5 µM (Magenta square); LEDGF 402–411 (Black full circle). All other experimental conditions are described in Materials and Methods.

The results depicted in Fig 8 clearly indicate that the observed stimulation by the INrs is not due to increases in either virus adsorption or reverse-transcriptase activity. Fig 8A and B demonstrate that the reverse transcription step was not effected by the addition of the INr peptides. This view is further supported by the kinetics studies described in Fig 8C. Dextran sulfate, various peptides and AZT were added at different times to HIV-1 infected Sup-T1 cells and the amount of viral P24 protein—reflecting efficiency of virus infection—was estimated at 48 h PI. As expected, dextran sulfate—which inhibits virus absorption [40]–[42]—blocked virus infection only when added during the first 4 h PI. AZT—which inhibits the viral reverse transcriptase [43]—blocked infection when added within the first 10 h of infection (Fig 8C). To reveal the time period during which the integration process occurs, LEDGF-derived peptide (aa 402–411) which has been shown to block viral IN enzymatic activity *in vitro* and *in vivo* [36] was used as a marker. It is clear that the LEDGF-derived peptide exerted its inhibitory effect up to about 18 h PI. During this time, the INr peptides showed a clear stimulatory effect which terminated shortly after the “integration period” (between 6 and 18 h PI).

**Figure 9:**
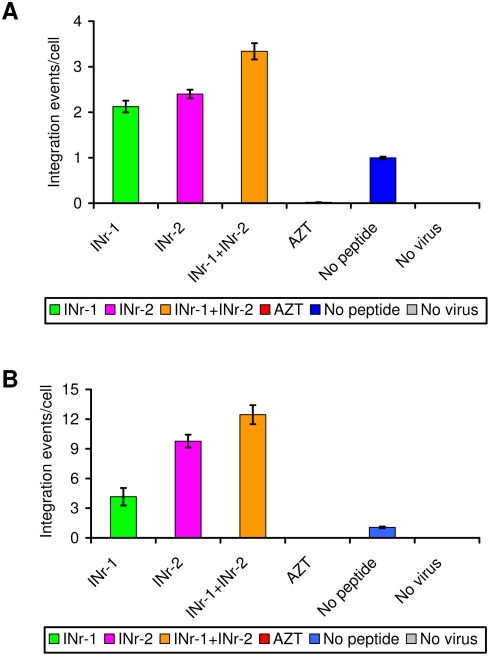
Stimulation of viral DNA integration events by the INr peptides. H9 T-lymphoid cells were incubated with the indicated peptides (12.5 µM) or AZT (2 µM) and following HIV-1 infection, the integrated viral DNA/cell was assessed by real-time PCR following: (A) a single cycle of infection at a MOI of 5 of Delta-env/VSV-G HIV-1 or (B) 8 days PI at a MOI of 0.01 of wild-type HIV-1. All other experimental conditions are described in Materials and Methods and in Ref. [22].

INr stimulation of the integration process was also observed when insertion of the viral DNA into the host DNA was estimated directly. As can be seen (Fig 9A and B), a ca. 2- to 2.5-fold stimulation of integration per cell was obtained following a single cycle of viral infection, while between 5- and 10-fold stimulation of integration was observed 8 days PI. Again, in both cases, integration stimulation was slightly enhanced when a mixture of the INr peptides was added to the cells.

## Discussion

It has been well established that following reverse transcription of the retroviral genome, the transcribed cDNA is integrated into the host chromosomal DNA, a process which is catalyzed by the viral IN enzyme. The integration target sites within the chromosomal DNA vary among different members of the Retrovirus family [44]. Integration of the HIV genome appears to favor active genes, while that of MuLV shows a strong bias for the transcription start sites and that of Avian sarcoma-leukosis virus (ASLV) does not exhibit any preference in its random integration into the host genome [44]. The number of integration events per cell also varies between different members of the retroviruses. Quantitative estimation has revealed that in cells infected by MuLV, there are 9 to14 detectable proviruses per individual host genome [18], [19], while in cells transformed by RSV, four to six integration events per haploid genome have been observed [20]. As many as 30 integration events per genome have been estimated in cells transformed by the Salmon swim bladder sarcomas retrovirus (SSSV) [45]. In contrast, in the case of HIV-1, which belongs to the lentiviruses (a subgroup of the Retrovirus family) [46], the number of integration events per genome is much more limited, apparently not exceeding two integration events per cell [16]. Since in practice, a relatively high number of lentiviral genome copies are available for integration, this low number of integration events is unexpected [16], [28], [29]. It has been reported that more than 20 reversed-transcribed cDNA molecules are present in each infected cell, most of which remain unintegrated [16], [28], [29]. Thus it appears that the large majority of the viral cDNA is prevented from integration. It may thus be speculated that while the IN of HIV-1 is subjected to a controlled-inhibition process, that of viruses such as MuLV or RSV is not.

Our present results clearly show that IN-derived peptides, which by themselves have no effect on IN enzymatic activity *in vitro*, greatly stimulate the integration of viral DNA in HIV-1 infected cells. Using an *in-vitro* integration assay system, we show that these peptides, namely INr-1 and INr-2, abrogate inhibition of integrase activity by the Rev protein as well as by the Rev-derived peptides. On the other hand, the INr peptides failed to abrogate the inhibitory effects of two LEDGF derived peptides [36], strengthening the view that the abrogation is due to specific interactions of the INr peptides with the Rev protein or Rev derived peptides. Specificity of interaction is further emphasized by our results showing that while the two INr peptides abrogated the inhibitory effect of Rev, INr-1 was able to abolish only the inhibitory effect of Rev 53–67, while INr-2 only that of Rev 13–23. Being cell permeable, the two INr peptides were able to abrogate the inhibitory effect exerted by the Rev peptides on the degree of virus infection. Furthermore both INr peptides enhanced virus infection in three different assay systems. Thus, the combination of the *in-vitro* and *in-vivo* (in cultured cells) experiments raise the interesting possibility that the limited number of integration events per cell may result from the interaction between the viral IN and the Rev protein, resulting in inhibition of HIV-1 IN activity. The INr peptides – as shown here – promote the dissociation of the IN-Rev complexes, thus allowing IN to be fully active and enhancing the integration process. Our results clearly show that incubation with the INr peptides stimulate viral DNA integration leading to multiple integration events. Thus these data further support the view that intracellularly, the viral Rev protein may inhibit/regulate IN activity and release from such inhibition may leads to stimulation of IN activity and multiple viral DNA integration events.

Recent results obtained in our laboratory, using various virus strains and different cell lines strongly support the suggested regulatory mechanism namely inhibition of viral DNA integration by the viral Rev protein (not shown and will be published elsewhere).

It is our view that the cell death and syncytia formation observed following infection of INr treated cells results from numerous integration events observed in these cells. The fact that a Rev analogue protein is absent from retroviruses, in which a high number of integration events per cell has been observed, further supports our present assumption that the viral Rev protein controls/regulates the integration process in HIV-1 infected cells.

Experiments are underway in our laboratory to better characterize the regulation of IN activity, especially by following the IN-Rev interaction in virus-infected cells.

## Materials and Methods

### Protein expression and purification

Expression and purification of histidine-tagged Rev-GFP were performed as described previously [34]. The histidine-tagged IN expression vector was a generous gift from Dr. A. Engelman (Department of Cancer Immunology and AIDS, Dana-Farber Cancer Institute, Division of AIDS, Harvard Medical School, Boston, Massachusetts, United States of America) and its expression and purification were performed essentially as described in Jenkins et al. [47].

### Mammalian cultured cells

Monolayer adherent HeLa, HEK293T, LC5-RIC and HeLa MAGI cells (TZM-bl) [48] expressing the β-galactosidase gene under regulation of the transactivation response element [49] were grown in Dulbecco's Modified Eagle's Medium (DMEM). The T-lymphocyte cell lines Sup-T1 and H9 were grown in RPMI 1640 medium. Cells beside the LC5-RIC cells were provided by the NIH Reagent Program, Division of AIDS, NIAID, NIH, USA and were incubated at 37°C in a 5% CO_2_ atmosphere. All media were supplemented with 10% (v/v) fetal calf serum, 0.3 g/l L-glutamine, 100 U/ml penicillin and 100 U/ml streptomycin (Biological Industries, Beit Haemek, Israel).

### Peptide synthesis, labeling and purification

Peptides were synthesized on an Applied Biosystems (ABI) 433A peptide synthesizer. For cellular-uptake studies, the peptides were labeled with fluorescein at their N terminus [50]. The peptides were also labeled with Trp at their N terminus for UV spectroscopy. Peptide purification was performed on a Gilson HPLC using a reverse-phase C8 semi-preparative column (ACE, advanced chromatography technologies, USA) with a gradient from 5% to 60% acetonitrile in water (both containing 0.001% v/v trifluoroacetic acid). Peptide concentrations were determined using a UV spectrophotometer (Shimadzu Kyoto, Japan)as described previously [51].

### Viruses

Wild-type HIV-1 was generated by transfection [52] of HEK293T cells with pSVC21 plasmid containing the full-length HIV-1_HXB2_ viral DNA [53]. Wild-type and Δenv/VSV-G [54] viruses were harvested from HEK293T cells 48 and 72 h post-transfection with pSVC21 Δenv. The viruses were stored at −75°C.

### ELISA-based binding assays

Protein-peptide binding was estimated using an ELISA-based binding assay exactly as described previously [55]. Briefly, Maxisorp plates (Nunc) were incubated at room temperature for 2 h with 200 µl of 10 µg/ml synthetic peptide in carbonate buffer. After incubation, the solution was removed, the plates were washed three times with PBS, and 200 µl of 10% BSA (Sigma) in PBS (w/v) was added for 2 h at room temperature. After rewashing with PBS, Rev-GFP, GFP alone or biotinylated-BSA-peptide conjugates (dissolved in PBS containing 10% BSA to give the appropriate concentrations (see legends to figures)), were added for further incubation for 1 h at room temperature. Following three washes with PBS, the concentration of bound biotinylated molecules was estimated after the addition of streptavidin-horseradish peroxidase (HRP) conjugate (Sigma), as described previously [56]. The concentration of bound protein molecules was estimated after the addition of anti-GFP mouse antibody (Santa Cruz) which then was interacted with rabbit anti-mouse IgG antibody conjugated to HRP. The enzymatic activity of HRP was estimated by monitoring the product's optical density (OD) at 490 nm using an ELISA plate reader (Tecan Sunrise). Each measurement was performed in duplicate.

### Determination of integrase activity

The IN enzymatic activity assay was performed using a previously described assay system [57], [58]. Briefly, the oligonucleotide substrate consisted of one oligo (5′-ACTGCTAGAGATTTTCCACACTGACTAAAAGGGTC-3′) labeled with biotin at the 3′ end and the other oligo (5′- GACCCTTTTAGTCAGTGTGGAAAATCTCTAGCAGT-3′) labeled with digoxigenin at the 5′ end. When inhibition was studied, the IN was preincubated with the peptide or protein for 15 min prior to addition of the DNA substrate. The entire IN reaction was followed by immunosorbent assay on avidin-coated plates as described previously [22], [58].

### Cell-penetration experiments

Fluorescein-labeled peptides at a final concentration of 10 µM in PBS were incubated with HeLa cells for 1 h at 37°C. After three washes in PBS, cells were visualized by a confocal microscopy as described previously [22].

### Effect of peptides on cell viability using the MTT (3-(4,5-dimethylthiazol-2-yl)-2,5-diphenyltetrazolium bromide) assay

Following incubation of the cells with the indicated peptides, the medium was removed and the cells were further incubated in Earl's solution containing 0.3 mg/ml MTT for 1 h. Subsequently, the solution was removed and the cells were dissolved in 100 µl DMSO for 10 min at room temperature. The DMSO-solubilized cells were transferred to a 96-well ELISA plate and OD values were monitored at a wavelength of 570 nm.

### HIV-1 titration by Multinuclear Activation of a Galactosidase Indicator (MAGI) assay

Quantitative titration of HIV-1 was carried out using the MAGI assay, as described by Kimpton and Emerman [49]. Briefly, TZM-b1 cells were grown in 96-well plates at 1×10^4^ cells per well and following 12 h incubation at 37°C, peptides were added; after an additional 2 h of incubation, the cells were infected with 50 µl of serially diluted virus (HIV-1 Δenv/VSV-G or wild-type HIV-1 which was obtained from infected lymphocytes every 2 days) as described [49]. Two days post-infection (PI), cultured cells were fixed and β-galactosidase was estimated exactly as described previously [49]. Blue cells were counted under a light microscope at 200× magnification.

### Infection of cultured lymphocyte cells with HIV-1

Cultured lymphocytes (1×10^5^) were centrifuged for 5 min at 2000 rpm and after removal of the supernatant, the cells were resuspended in 0.2 to 0.5 ml of RPMI 1640 medium containing virus at a multiplicity of infection (MOI) of 0.01 and 5. Following absorption for 2 h at 37°C, the cells were washed to remove unbound virus and then incubated at the same temperature for an additional 1 to 8 days [22].

### Quantitative estimation of HIV-1 infection by determination of extracellular p24

Lymphoid cells were incubated with the indicated peptides for 2 h and following infection with wild-type HIV-1 at a MOI of 0.01 (as described above), the cells were incubated for 8 days, or 48 h at a MOI of 1. The amount of p24 protein was estimated in the cell medium every 2 days exactly as described previously [22].

### PCR analysis of early viral genes

Sup-T1 cells were incubated with 12.5 µM of peptides or with 2 µM azidothymidine (AZT) for 2 h and then were infected with HIV-1 Δenv/VSV-G virus at a MOI of 2, and further incubated for 6 h . The viral Gag or Nef DNA sequences were amplified using specific primers: Gag-specific primers, 5′-AGTGGGGGGACATCAAGCAGCCATG-3′ and 5′-TGCTATGTCAGTTCCCCTTGGTTCTC-3′, and Nef-specific primers, 5′-CCTGGCTAGAAGCACAAGAG-3′ and 5′-CTTGTAGCAAGCTCGATGTC-3′. The fragments were amplified from 10 ng of total cell DNA in a 25-µl reaction mixture containing 1× PCR buffer, 3.5 mM MgCl_2_, 200 µM dNTPs, 300 nM primers, and 0.025 units/µl of *Taq* polymerase. The PCR conditions were as follows: a DNA denaturation and polymerase activation step of 5 min at 95°C and then 29 cycles of amplification (95°C for 45 s, 60°C for 30 s, 72°C for 45 s).

### Time-of-addition assay (the time at which various components were added on virus infected cultured cells)

Sup T1 cells were infected with wild-type HIV-1 at a MOI of 2, and the test compounds were added at different time points after infection (0, 2, 4,…, 24 h). Viral p24 production was determined at 48 h PI [59]. Dextran sulfate was tested at 20 µM, AZT at 2 µM, LEDGF 402-411, INr-1 and INr-2 at 12.5 µM.

### Quantitative analysis of the copy numbers of HIV-1 DNA integrated into cellular genome

The integration reaction was estimated essentially as described previously [22]. Briefly, following incubation of the indicated peptides with H9 or Sup-T1 cells for 2 h, the cells were infected with a HIV-1 Δenv/VSV-G virus at a MOI of 5 (as described above) for 24 h or with wild-type HIV-1 at a MOI of 0.01 for 8 days. Integrated HIV-1 sequences were amplified by two PCR replication steps using the HIV-1 LTR-specific primer (LTR-TAG-F 5′-ATGCCACGTAAGCGAAACTCTGGCTAACTAGGGAACCCACTG-3′) and Alu-targeting primers (first-Alu-F 5′-AGCCTCCCGAGTAGCTGGGA-3′ and first-Alu-R 5′-TTACAGGCATGAGCCACCG-3′) [60]. Alu-LTR fragments were amplified from 10 ng of total cell DNA in a 25-µl reaction mixture containing 1× PCR buffer, 3.5 mM MgCl_2_, 200 µM dNTPs, 300 nM primers, and 0.025 units/µl of *Taq* polymerase. The first-round PCR cycle conditions were as follows: a DNA denaturation and polymerase activation step of 10 min at 95°C and then 12 cycles of amplification (95°C for 15 s, 60°C for 30 s, 72°C for 5 min).

During the second-round PCR, the first-round PCR product could be specifically amplified by using the tag-specific primer (tag-F 5′-ATGCCACGTAAGCGAAACTC-3′) and the LTR primer (LTR-R 5′-AGGCAAGCTTTATTGAGGCTTAAG-3′) designed by PrimerExpress (Applied Biosystems) using default settings. The second-round PCR was performed on 1/25th of the first-round PCR product in a mixture containing 300 nM of each primer, 12.5 µl of 2× SYBR Green master mixture (Applied Biosystems) at a final volume of 25 µl, run on an ABI PRIZM 7700 (Applied Biosystems). The second-round PCR cycles began with DNA denaturation and a polymerase-activation step (95°C for 10 min), followed by 40 cycles of amplification (95°C for 15 s, 60°C for 60 s).

For generation of a standard calibration curve, the SVC21 plasmid containing the full-length HIV-1_HXB2_ viral DNA was used as a template. In the first-round PCR, the LTR-TAG-F and LTR-R primers were used and the second-round PCR was performed using the tag-F and LTR-R primers. The standard linear curve was in the range of 5 ng to 0.25 fg (*R* = 0.99). DNA samples were assayed with quadruplets of each sample. For further experimental details see ref [22]. The cell equivalents in the sample DNA were calculated based on amplification of the 18S gene by real-time PCR as described in [61].

### Determination of HIV-1 infection by the LC5-RIC assay

LC5-RIC cells is a HeLa-derived cell line that contains a retroviral vector encoding the human CD4 gene [62] and the Rev-inducible reporter construct pLRed2xINSR, (described in [25], [38], [39]).

Cells were kept under standard cell culture conditions using Dulbecco's Modified Eagle Medium with 10% fetal calf serum (FCS) and 2 mM Glutamax I (Invitrogen, Karlsruhe, Germany). For primary infection assays, LC5-RIC cells were seeded at a density of 1.4×10^4^ cells per well in 96-well plates (Falcon). Peptide stock solutions were prepared by dissolving peptides in 80% DMSO, 20% H_2_O. Twenty four hours later, serial dilutions of peptide stock solutions prepared in fresh cell culture medium were added to each well. Final peptide concentrations in each well are indicated in Fig 6. In addition, wells were treated with serial dilutions of the solvent lacking the peptide to control for solvent effects. After 30 minutes pre-incubation, supernatant from the HIV-1 producer cell line KE37/1-IIIB [63] containing 5×10^7^ RNA copies of HIV-1 was added to each well. Three wells were incubated with supernatant from uninfected KE37/1 cells as negative controls. Cells were incubated for the time periods indicated in Fig 6 at 37°C, washed twice with PBS and red reporter fluorescence was estimated in a microplate fluorimeter (ThermoFisher).

All the results described in the present study are averages of at least three–four determinations, where the standard deviation never exceeded ±20%.

Error bars were calculated using standard deviation function of Microsoft Excel ™.
